# Interactive AI annotation of medical images in a virtual reality environment

**DOI:** 10.1007/s11548-025-03497-9

**Published:** 2025-08-18

**Authors:** Lotta Orsmaa, Mikko Saukkoriipi, Jari Kangas, Nastaran Rasouli, Jorma Järnstedt, Helena Mehtonen, Jaakko Sahlsten, Joel Jaskari, Kimmo Kaski, Roope Raisamo

**Affiliations:** 1 Faculty of Information Technology and Communication Sciences, Tampere University, Kalevantie 4, 33014 Tampere, Finland; 2 Department of Computer Science, Aalto University School of Science, Otakaari 1B, 00076 Espoo, Finland; 3 Department of Radiology, Tampere University Hospital, Wellbeing Services County of Pirkanmaa, Elämänaukio 2, 33520 Tampere, Finland; 4 Faculty of Medicine and Health Technology, Tampere University, Kalevantie 4, 33014 Tampere, Finland

**Keywords:** Medical imaging, AI annotation, Virtual reality, Interactive AI

## Abstract

****Purpose**:**

Artificial intelligence (AI) achieves high-quality annotations of radiological images, yet often lacks the robustness required in clinical practice. Interactive annotation starts with an AI-generated delineation, allowing radiologists to refine it with feedback, potentially improving precision and reliability. These techniques have been explored in two-dimensional desktop environments, but are not validated by radiologists or integrated with immersive visualization technologies. We used a Virtual Reality (VR) system to determine whether (1) the annotation quality improves when radiologists can edit the AI annotation and (2) whether the extra work done by editing is worthwhile.

****Methods**:**

We evaluated the clinical feasibility of an interactive VR approach to annotate mandibular and mental foramina on segmented 3D mandibular models. Three experienced dentomaxillofacial radiologists reviewed AI-generated annotations and, when needed, refined them at the voxel level in 3D space through click-based interactions until clinical standards were met.

****Results**:**

Our results indicate that integrating expert feedback within an immersive VR environment enhances annotation accuracy, improves clinical usability, and offers valuable insights for developing medical image analysis systems incorporating radiologist input.

****Conclusion**:**

This study is the first to compare the quality of original and interactive AI annotation and to use radiologists’ opinions as the measure. More research is needed for generalization.

## Introduction and background

Interpreting radiological images, such as Computed Tomography (CT), Cone Beam Computed Tomography (CBCT), and Magnetic Resonance Imaging (MRI), is essential for diagnosis and treatment planning [1]. The segmentation of radiological images and annotation of specific anatomical structures observable in the image are important analysis phases. The image annotation is done manually, which is time-consuming. A further challenge of annotation is its subjective essence [2].

The problems of image annotation have motivated the development of radiological AI annotation [3]. When properly trained and validated, AI can offer high precision, efficiency, and reliability reducing manual labor [4]. However, AI annotation without user input often lacks robustness [5], as radiologists may disagree.

User-initiated corrections allow the interactive AI to add or remove areas from the annotation. This is connected to contestability, one of the attributes linked to eXplainable Artificial Intelligence (XAI). XAI is essential in the medical field, as it clarifies the logic behind the algorithm to the user, making AI more transparent [6], and allows clinicians to determine whether to trust a decision. XAI helps radiologists grasp the reasoning behind results, addressing some ethical concerns in medical AI. With XAI, the radiologists could query the AI about specific annotation areas and request corrections.

In interactive annotation, human feedback is used to refine the initial annotation results. One of the most used techniques is click-based interaction [7], wherein an expert locates and selects erroneously predicted voxels and assigns the correct label to each. The interactive annotation method then updates the entire annotation based on this user-provided correction [8]. A single click-based interaction can then correct a large erroneous annotation.

High-fidelity 3D models can be created from 2D radiological images [9], allowing users to explore all image elements, including underlying structures. 3D medical images improve spatial understanding of structures compared to 2D images [10]. Conventionally, image analysis is performed through DICOM monitors, which limits interaction with the images [11].

Virtual Reality (VR) has been studied in medicine for its potential to enhance decision-making, education, and patient care [12]. VR offers an immersive platform for analyzing 3D medical images due to intuitive manipulation [13] and increased interaction methods [14].

We developed a VR system to analyze AI-generated 3D annotation of the mandibular and mental foramina. An interactive AI algorithm allowed users to add or remove specific voxels. We evaluated whether clinician input improves the original AI annotation. Two of the three participating radiologists rated the interactive annotation significantly higher than the original but also found that the usability of the VR system was lower with the interactive annotation. The study advances the exploration of AI and VR in annotation analysis, focusing on the impact of user input.

The paper is organized as follows: Sect. 2 details the AI algorithm and VR system used. Section 3 covers the experimental methods, including participants, tasks, conditions, and design. Finally, the results are presented in Sect. 4 and discussed in Sect. 5.

## Apparatus

### VR software

The experiment software for the VR environment was developed using the Unity Game Engine, the OpenXR Plugin [15], and the Unity Volume Rendering Plugin [16]. We used direct volume rendering to visualize medical image scans within the game engine.

**Fig. 1:**
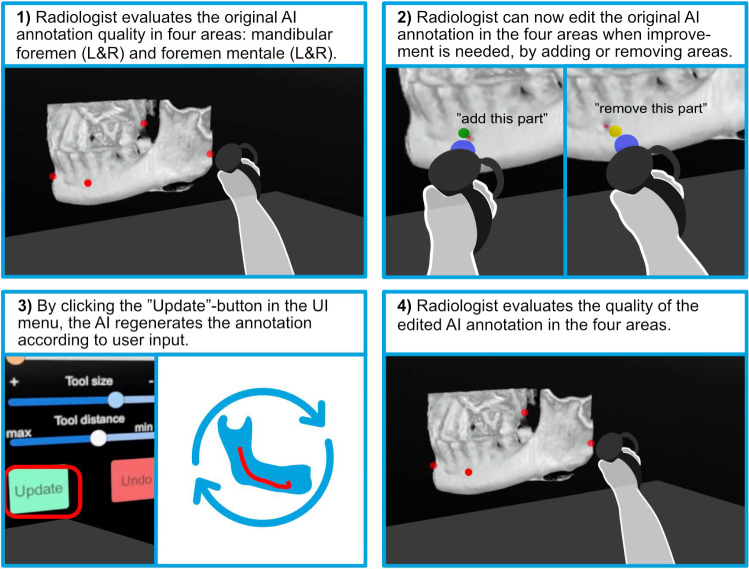
The AI-generated annotation quality assessment involves four stages. (1) Reviewing the original AI annotation in four areas (left/right mandibular foramen and left/right mental foramen). (2) Editing the annotation by instructing the AI to add or remove areas, using an indicator (blue) to show the editing location. (3) Clicking “Update” to regenerate the annotation. (4) Re-evaluating the edited annotation

We developed an interactive marking tool (see Fig. 1) that lets users annotate 3D image models using a handheld controller. By pressing buttons (A or B for left/right mouse clicks), users place colored spheres at desired locations. These markers remain attached to the image during transformations like rotation or repositioning. Marker coordinates and types are logged in a database for subsequent AI analysis, facilitating precise and intuitive 3D annotations.

The user has access to a menu containing various elements: two sliders to adjust the visibility of the segmented 3D jaw model and annotations. In the Edit session, the menu expands to include two additional sliders to adjust the size of the sphere and its distance from the controller along its beam. An “Update” button imports the latest AI-generated segment based on the user’s edits. The scenes contain two more objects for user interaction: a plane for cutting the volume and a box that interacts with the volume to cut its inner part.

### Dataset

The deep learning model was trained on a dataset of 982 CBCT scans, as introduced in [17]. For evaluation, a subset of 50 clinically heterogeneous scans from a holdout test set was used from [18]. No new data were collected or annotated for this experiment. Scans were preprocessed to a $0.4~\text {mm}^3$ isotropic resolution. In both datasets, the mandibular canal is annotated with centerline coordinates, connected into a spline, and dilated into a 1.5-mm-diameter tube to approximate its segmentation, used as ground truth for training and evaluation.

The mandibular canal was selected to align with the dentomaxillofacial radiology expertise of radiologists in virtual reality (VR) experiments. The evaluation subset, comprising 50 scans from 44 patients, was selected from a set of 120 normal, orthognathic, or postoperative orthognathic scans. Selection relied on two criteria: the lowest Dice scores from the deep learning model without interactions and minimizing scans per patient. Only imperfect segmentations permit improvement through radiologist edits in virtual reality (VR). Poor segmentations were thus chosen to test annotation quality improvements. This approach evaluates the clinical value of editing efforts. A separate patient scan was used for tutorial and training purposes.

### Interactive annotation

The click-based interactive annotation model *2 S-ICR*, as introduced in [19], was employed in this study. The method proposes an initial segmentation map, which can then be refined with individual clicks with either positive or negative voxels. The synthetic interaction events are generated by sampling clicks exclusively from regions containing false positives or false negatives, identified by comparing the annotation output with the ground truth. The synthetic sampling method was modified by excluding boundary areas from the interaction events as the boundary region is not accurately defined with the used dataset. The hyperparameter $p$ for dropping the previous mask was found optimal at $p = 0.6$ in this dataset.

For the 2 S-ICR model training, the data were preprocessed by clipping voxel intensity values to [$-1000$, 3095] Hounsfield units, followed by rescaling the intensity values to the range of [0, 1]. The data were augmented with random spatial and intensity transformations. The spatial transformations included flips, rotations in the range of $[-90^{\circ }$, $90^{\circ }$], and translations of up to 16 pixels across all spatial dimensions, which were applied with a probability of 0.5. The intensity transformations included changes in the gamma value in the range of [0.5, 1.5], offsets up to 0.1, Gaussian noise, and Gaussian smoothing, each applied with a probability of 0.25. The model was trained for 300 epochs, and the model weights were learned with the Adam optimizer [20].

The 2 S-ICR model interfaces with the VR system through a shared database, where user interactions are stored as 3D coordinates and interaction types (positive or negative voxels). When a user adds an interaction via the VR marking tool, the DL model automatically updates the segmentation upon detecting the new event in the database. The updated segmentation is visualized in the VR environment within a few seconds, providing a near-real-time experience.

Annotation performance is evaluated using the Dice similarity coefficient (DSC). The DSC is a measure of volumetric overlap that is calculated as follows:

1 $$\begin{aligned} \text {DSC}(\boldsymbol{y},\hat{\boldsymbol{y}}) = 2\frac{\sum _{i,j,k} \boldsymbol{y}_{i,j,k}\hat{\boldsymbol{y}}_{i,j,k}}{\sum _{i,j,k} \boldsymbol{y}_{i,j,k} + \sum _{i,j,k} \hat{\boldsymbol{y}}_{i,j,k}}, \end{aligned}$$

where $\boldsymbol{y}$ is a target volume and $\hat{\boldsymbol{y}}$ is the corresponding binary segmentation volume generated by thresholding the model output with a value of 0.5.

## Methods

### Participants

Three dentomaxillofacial radiologists participated in the study. One identified as female and two as male. The mean age of the experts was 47 years. Radiologist A had 40 years of experience in dentomaxillofacial radiology, while Radiologists B and C each had 8 years of experience. All experts were right-handed and had extensive VR experience or tried VR more than a few times. Two of the radiologists, Radiologists A and B, had previously contributed to annotating the dataset used in this study. Specifically, Radiologist A annotated both test and training data, while Radiologist B annotated training data.

All experts signed a consent form before the experiment. The participation was not compensated.

### Procedure

#### Before the experiment

The radiologists were asked to read the subject information sheet, sign an informed consent form, and complete a background questionnaire. The facilitator read out loud the general description script.

#### Experimental task

Each radiologist watched a video of the VR system and participated in a practice session for its use.

There were 50 AI-segmented lower jaw CBCT models for each radiologist to study. In each task, the radiologist had to analyze the AI-generated 3D annotations of the mandibular foramen and foramen mentale, on the right and left sides, making a total of 192 annotation analysis tasks.

Each model was analyzed in both “Observe” and “Edit” conditions. In “Observe,” the radiologist could not give feedback to the AI. In “Edit,” the radiologist could order the AI to remove areas of the annotation or add new areas, after which the AI would regenerate the annotation.

Being ready with their analysis, the radiologist gave a score on a scale of $[0 - 4]$ of the annotation quality (0: Not usable for diagnostics, (1) partly usable for diagnostics, canal visibility below one half, (2) minor issues with almost fully usable for diagnostics, (3) almost perfect, (4) perfect) [18]. The radiologist would then answer a UMUX-LITE usability questionnaire [21] regarding the VR system with the given condition.

Then, the radiologist pressed the Next button again to start another task. When all tasks were completed, pressing the Next button ended the experiment.

#### After the experimental tasks

The radiologists were interviewed about their experiences. The structured interview had three sections: manipulation methods (“How did you use direct manipulation and locomotion?”, “What do you think of the methods?”), volume editing tools (“What is your opinion of the volume editing tools (Sliders, Slicing)?”), and AI (“What is your opinion of the AI-generated annotations?”, “How understandable are the annotations?”, “What do you think of the AI-generated annotation editing tools?”).

### Experiment design

The experiment followed a 2 $\times$ 1 within-subjects design, with the independent variable being the interaction method with two levels: (1) Observe and (2) Edit. The conditions were counterbalanced by switching their order with each radiologist (Radiologist A: 12/21/12/21...; Radiologist B: 21/12/21/12...). The dependent variables included subjective ratings of annotation quality and system usability.

## Results

We first present the quantitative results (Sect. 4.1) in three subsections: the results of the annotation quality evaluations done by radiologists (Sect. 4.1.1), AI performance rating (Sect. 4.1.2), and usability rating (Sect. 4.1.3). Then we present the qualitative results (Sect. 4.2).

### Quantitative results

#### Annotation quality evaluation

**Fig. 2:**
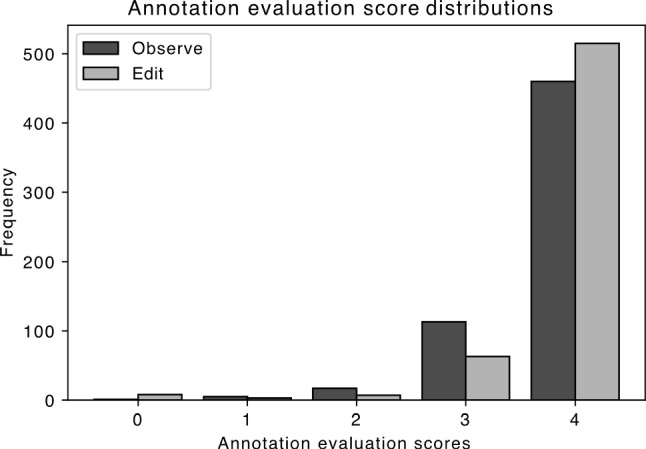
Comparison of the annotation evaluation score distributions. The highest score values are more frequent in the Edit condition

The distributions of the annotation evaluation score values are shown in Fig. 2. In both conditions, the highest values are in the majority, but increased in the Edit condition.

**Fig. 3:**
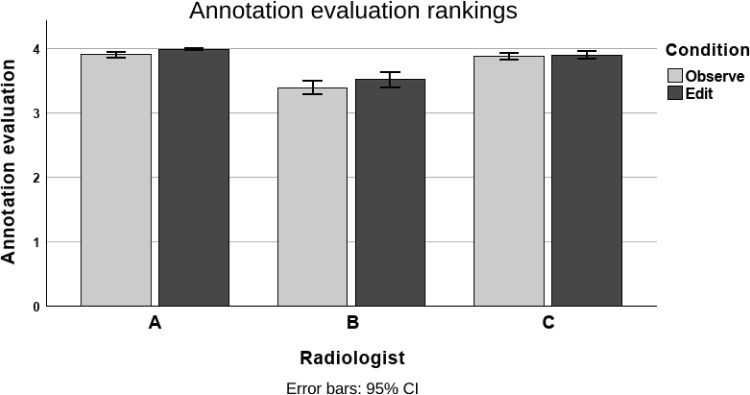
Annotation evaluation scores for each radiologist, with both conditions

The annotation evaluation results for the Observe and Edit conditions are shown in Fig. 3. The paired sample sign test was used as the Wilcoxon Signed-rank test cannot be used with ordinal data. The test showed that the annotation quality was significantly higher in the Edit condition than in the Observe condition for Radiologists A $p < 0.001$ and B $p < 0.001$. The median score was 4 in both conditions among the two radiologists. There were no statistical differences in the conditions with Radiologist C.

We also analyzed inter-observer agreement through pairwise comparisons as shown in Table 1. Each radiologist pair received a higher agreement rate in annotation quality in the Edit condition compared to the Observe condition.

#### Annotation performance in “Observe”

The results of the synthetic interaction performance for the 50 selected 3D-CT cases are presented in Table 2.

#### Usability rating

**Fig. 4:**
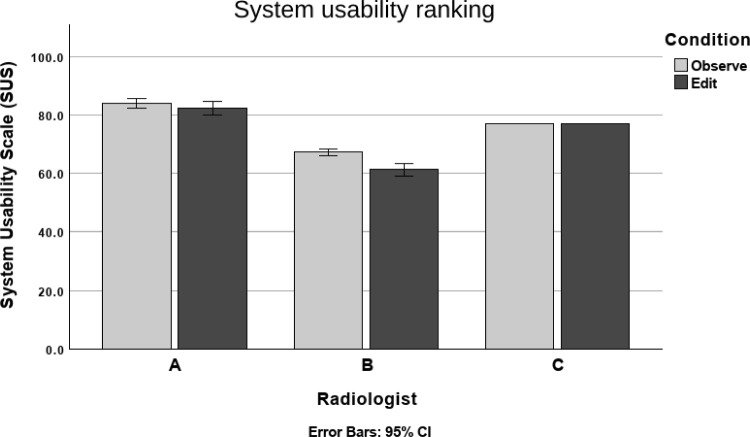
Usability evaluation scores for each radiologist, with both conditions

Final scores for the UMUX-LITE questions were obtained by calculating the average of the two scores [21] and converted to the SUS scale using the formula.

2 $$\begin{aligned} \text {UMUX-LITE}&= 0.65 \times \frac{(\text {UMUX}_1 + \text {UMUX}_2 - 2) \times 100}{12} \nonumber \\&+ 22.9, \end{aligned}$$

where $\hbox {UMUX}_1$ and $\hbox {UMUX}_2$ are the UMUX-LITE scores. UMUX-LITE covers a narrower version of the SUS scale $(22.9-87.9)$. The usability evaluation scores are shown in Fig. 4.

**Table 1:** The inter-observer agreement rates between radiologist pairs

Radiologist pair	A–B (%)	A–C (%)	B–C (%)
Observe	54.1	83.2	55.6
Edit	66.3	93.4	65.3

The first number in each column shows the inter-observer agreement rate in the Observe condition, and the second number shows the agreement rate in the Edit condition. In the Edit condition, the agreement rates are consistently higher

**Table 2:** Model segmentation performance with synthetic interaction events on the evaluation subset ($n=50$), against approximate mandibular canal ground truth, as described in the Dataset Sect. 2.2

Repeat	0 Click	1 Click	5 Clicks	10 Clicks	0 to 10 Avg
1	0.490 ± 0.120	0.510 ± 0.115	0.569 ± 0.092	0.645 ± 0.081	0.569 ± 0.109
2	0.490 ± 0.120	0.510 ± 0.120	0.574 ± 0.093	0.645 ± 0.077	0.571 ± 0.109
3	0.490 ± 0.120	0.510 ± 0.114	0.568 ± 0.094	0.646 ± 0.080	0.570 ± 0.110

The mean ± standard deviation of DSC is documented for the reported interaction points and on average for all interaction events

The UMUX-LITE scores and converted SUS scores were tested for normality with Shapiro–Wilk. The normality assumption was rejected, and the Wilcoxon signed-rank test was used to test both the UMUX-LITE scores and converted SUS scores for significance. As the significance was the same $(p < 0.05)$, we present the results of the converted SUS scores for comparison.

The Wilcoxon signed-rank test showed that the SUS score was significantly higher in the Observe condition than in the Edit condition with Radiologists A $(Z = -2.005, p < 0.05)$ and B $(Z = -4.658, p < 0.05)$. The median score on a 7-point scale item was 77.1 in both conditions.

### Qualitative results

Radiologists’ answers were gathered and sorted into similar groups for qualitative analysis and then thematically analyzed based on the stages in Braun and Clarke [22]. Relevant answers were coded, sorted into different themes, described, and revised. Four final themes formed were: (1) “Unintuitive image manipulation,” (2) “Task load,” (3) “Positive aspects of the VR system (excl. AI),” and (4) “Opinions regarding AI features”.

#### Unintuitive image manipulation

The unintuitivity of the image manipulation related to the locomotion and direct object manipulation methods. Problems in direct object manipulation caused the experts to rely on Diving instead, at least sometimes. Radiologist A commented:

For getting closer to specific areas, Diving was necessary for usability reasons (even though I wanted to use direct object manipulation).

Regarding Diving, Radiologists A and B addressed the unintuitiveness of the technique:

“I used direct object manipulation the most as it felt more natural.—Radiologist B”

“I did not find Diving logical, it should be developed so that the image is brought closer instead of the VR environment.—Radiologist A”

#### Task load

Regarding the factors contributing to task load, Radiologist A addressed that there was a lower task load when not needing to edit the AI annotations. However, Radiologist A also affirmed that image manipulation methods were needed to evaluate clinically difficult cases:

When there was a clinically difficult case (operated patient), I had to bring the image slightly closer and rotate it.

Finally, Radiologist A faced controller precision problems when trying to edit the AI-generated annotations, as the controllers lacked motion stability:

The VR controllers do not have motion stabilizer, so putting the markings into exact location was hard.

#### Positive aspects of the VR system (excl. AI)

Radiologists A and C found most of the interaction methods logical. Radiologist C also considered diving to be well-suited for their task:

I used the joystick frequently in navigation. I did not find the usability of the direct object manipulation method good enough. Therefore, I used Diving instead; it is better suited for a job that involves frequent sitting.

#### Opinions regarding AI features

Regarding AI features, all three radiologists felt that the annotations were generally well-made.

Radiologists B and C faced problems while correcting the AI-generated annotations. The annotation-adding tool was considered to be limited by Radiologist B, because they felt that by adding, they could only increase length and location but not width. Also, Radiologist B encountered the problem that the AI started editing annotation areas that were not in the direct area of the change:

“The AI annotations broke multiple times when the AI changed the annotation from the other side, even though it was not the location where the changes were directed.”

Radiologist C had the most problems with AI annotation editing due to general difficulties:

“The tools that allowed editing the AI-generated annotation were difficult to use. I cannot say that the tools did what I wanted them to do. In my mind, it was clear what I wanted to do, but it was not possible.”

## Discussion

In this study, we found a trade-off between annotation quality and usability in interactive AI. Editing the AI-generated annotations improved their quality and increased inter-observer agreement, but also decreased system usability.

All radiologists were satisfied with the results of the AI annotation. They were surprised that the AI could automatically annotate the nerve endings and openings of the mandibular canal with great results in clinically difficult cases. In most cases, editing the original AI-generated annotation was unnecessary. Although the study results point to a higher quality of the interactive AI annotation compared to the original, the results could be better, as in a few cases, two of the three radiologists saw the AI worsen the annotation in a non-targeted area. This explains the lowest ratings for the annotation quality in the Edit condition. More work should be done to improve the usability of editing to determine whether usability issues mask potential improvements in annotation quality.

Several technical factors also explain these shortcomings. First, although radiologists could set the radius of the interaction sphere in VR, only the center coordinates were passed to the annotation algorithm. This mismatch confused and reduced the perceived efficacy of localized edits. Second, the model was trained on splines of uniform canal width, making it difficult to handle real-world variability in canal thickness and to adjust the canal width based on user input. Third, the global editing approach caused local refinements to propagate throughout the volume, which sometimes confused radiologists trying to target specific regions. Finally, the model was optimized for the mandibular canal as a whole, while the clinical focus in this study was on the mental and mandibular foramina ends, creating a gap between training objectives and actual evaluation targets.

Addressing these issues requires coordinated improvements in the AI model and the user interface. Ensuring the sphere’s radius fully corresponds to a clearly defined volume of interest would better reflect radiologists’ intended refinements. Introducing more anatomically diverse training data, rather than relying on uniform spline interpolations, would allow the model to accommodate canal shape and thickness variations. Constraining edits to clearly demarcated local regions could prevent inadvertent updates elsewhere in the volume, and aligning the training objectives with the areas of clinical interest would likely enhance performance and consistency.

The findings on annotation quality and inter-observer agreement rate improvements seem to point in a similar direction as in earlier studies. Spaanderman et al. [23], a minimally interactive AI segmentation method received more overlap with radiologists’ reference annotation. Also, in Wei et al. [24], an interactive AI method substantially improved radiotherapy annotation accuracy. The results were found annotating 3–5 slices in one to three rounds.

This study is a step toward interactive AI annotation research that aims to consider the subjective analysis conducted by radiologists, a clinically used method. Although variables such as the DSC are necessary to determine the quality, they can differ from the subjective evaluations. As radiologists are the evaluators, human factors need to be studied from a user-centered perspective. We noticed that in some cases, the radiologists gave lower scores to the edited AI annotation than the original, which is thought to be because the AI could make unlocalized changes. These human factors issues cannot be found with technical analyses alone.

### Limitations

Our AI system occasionally modified areas beyond those intended by the radiologist for editing. Implementing spatial constraints could have mitigated some of the lower scores observed in the Edit condition. Although our study involved only three expert radiologists, we addressed this limitation by analyzing a substantial number of images per expert. Furthermore, the study focused exclusively on mandibular canal segmentation, chosen to align with the dentomaxillofacial radiology expertise of the participating radiologists, which may limit the generalizability of our findings to other anatomical regions. Nevertheless, the 2 S-ICR deep learning model and VR system show potential for adaptation to other regions through training with region-specific datasets, as evidenced in prior work [19], though this was not evaluated here. An additional limitation is the relatively low Dice similarity coefficients (DSC). These are partly due to approximate ground truth labels, generated from sparse splines and uniformly dilated, which introduce label noise. Moreover, the evaluation subset was chosen for low baseline segmentation performance, making improvements more meaningful but reducing overall scores. Despite this, prior work has shown that such annotations can still be clinically useful [17, 18]. Our framework prioritizes expert-in-the-loop correction over full automation, enabling radiologists to produce clinically acceptable results through efficient interaction. Thus, while Dice remains informative, it does not fully capture the practical value of this system.

### Future

Our findings indicate that interactive (contestable) AI-based annotation works for professional use. The AI results are clinically relevant and of satisfactory quality. In future studies, we will further study the benefits of explainability with 3D medical images in VR. We seek to enhance other XAI attributes beyond contestability (e.g., interpretability), which could increase trust in the system.

## Conclusion

Three experienced dentomaxillofacial radiologists evaluated AI-generated segmentations of the mandibular canal and mental foramina in 50 segmented 3D mandibular CT volumes. When needed, they refined the segmentations at the voxel level using a Virtual Reality (VR) interface with click-based input. We compared the quality and usability of fully automated segmentations versus those refined through expert-guided interactive segmentation. The findings show that expert input within an immersive VR environment can improve segmentation accuracy and clinical relevance. However, interactive refinement introduced usability challenges, underscoring the need for further user-centered interface development. To our knowledge, this is the first study to systematically compare unassisted and expert-refined segmentations in VR, incorporating radiologists’ subjective evaluations.
